# Anti-PD-1 Therapy Does Not Influence Hearing Ability in the Most Sensitive Frequency Range, but Mitigates Outer Hair Cell Loss in the Basal Cochlear Region

**DOI:** 10.3390/ijms21186701

**Published:** 2020-09-13

**Authors:** Judit Szepesy, Gabriella Miklós, János Farkas, Dániel Kucsera, Zoltán Giricz, Anita Gáborján, Gábor Polony, Ágnes Szirmai, László Tamás, László Köles, Zoltán V. Varga, Tibor Zelles

**Affiliations:** 1 Department of Pharmacology and Pharmacotherapy, Semmelweis University, H-1089 Budapest, Hungary; szepesy.judit@med.semmelweis-univ.hu (J.S.); miklosgala@gmail.com (G.M.); farkas.janos2@med.semmelweis-univ.hu (J.F.); kucsera.daniel@med.semmelweis-univ.hu (D.K.); giricz.zoltan@med.semmelweis-univ.hu (Z.G.); koles.laszlo@med.semmelweis-univ.hu (L.K.); varga.zoltan@med.semmelweis-univ.hu (Z.V.V.); 2 Department of Otorhinolaryngology, Head and Neck Surgery, Semmelweis University, H-1083 Budapest, Hungary; gaborjan@fulo.sote.hu (A.G.); polony@fulo.sote.hu (G.P.); szirmai.agnes@med.semmelweis-univ.hu (Á.S.); tamas.laszlo@med.semmelweis-univ.hu (L.T.); 3 HCEMM-SU Cardiometabolic Immunology Research Group, Semmelweis University, H-1089 Budapest, Hungary; 4 Pharmahungary Group, H-6722 Szeged, Hungary; 5 Department of Pharmacology, Institute of Experimental Medicine, H-1083 Budapest, Hungary

**Keywords:** immune checkpoint inhibitor, anti-PD-1 antibody, hearing loss, immune-related adverse events (irAE), auditory brainstem response (ABR), cochleogram, spiral ganglion neuron, Iba1, hair cells, macrophage

## Abstract

The administration of immune checkpoint inhibitors (ICIs) often leads to immune-related adverse events. However, their effect on auditory function is largely unexplored. Thorough preclinical studies have not been published yet, only sporadic cases and pharmacovigilance reports suggest their significance. Here we investigated the effect of anti-PD-1 antibody treatment (4 weeks, intraperitoneally, 200 μg/mouse, 3 times/week) on hearing function and cochlear morphology in C57BL/6J mice. ICI treatment did not influence the hearing thresholds in click or tone burst stimuli at 4–32 kHz frequencies measured by auditory brainstem response. The number and morphology of spiral ganglion neurons were unaltered in all cochlear turns. The apical-middle turns (<32 kHz) showed preservation of the inner and outer hair cells (OHCs), whilst ICI treatment mitigated the age-related loss of OHCs in the basal turn (>32 kHz). The number of Iba1-positive macrophages has also increased moderately in this high frequency region. We conclude that a 4-week long ICI treatment does not affect functional and morphological integrity of the inner ear in the most relevant hearing range (4–32 kHz; apical-middle turns), but a noticeable preservation of OHCs and an increase in macrophage activity appeared in the >32 kHz basal part of the cochlea.

## 1. Introduction

Activation of cellular immunity and abolishment of self-tolerance against tumor cells in the host organism (a.k.a., immune checkpoint inhibition, ICI) is an efficient and preferred way of antitumor pharmacotherapy with growing clinical applications and expanding therapeutic indications.

According to the cancer immunoediting hypothesis, one of the mechanisms tumor cells use to escape from immunosurveillance is to establish an immunosuppressive microenvironment by production of adenosine [1] or expressing programmed death-ligand 1 (PD-L1), a negative costimulatory molecule of the B7-CD28 superfamily. Binding of their PD-L1 to the programmed cell death protein 1 (PD-1) receptors on T-cells results in the inhibition of T-cell proliferation and cytokine production with the consequent evasion of immunosurveillance [2,3]. Inhibitors of the PD-1 receptor (like monoclonal antibodies such as pembrolizumab or nivolumab) are one option amongst the immunotherapeutic agents called checkpoint inhibitors that prevent the activation of these negative costimulatory pathways and thus restore the host’s anti-tumor immunity [4,5,6]. Numerous tumors, including advanced metastatic melanoma, colorectal cancer, non-small-cell lung cancer (NSCLC), and renal cell carcinoma (RCC), show promising responses to anti-PD-1 monoclonal antibody treatment [4,5,7,8].

However, activation of the immune system often causes atypical side effects, called immune-related adverse events (irAE). The immunological imbalance may lead to uncontrolled immune responses and breakdown of the peripheral tolerance, resulting in autoimmune-like symptoms. The most common irAEs involve gastrointestinal, dermatologic, hepatic, and endocrine toxicities. Less frequently, symptoms affecting the lung, eye, kidney, pancreas, the nervous, and the hematopoietic system are reported, as well [4,7,9,10,11].

Publications on cases of audiovestibular adverse events are much more sporadic [12,13,14] and no clinical studies have evaluated the impact of PD-1 inhibitors on the audiovestibular system so far. Taking into consideration that high-frequency hearing loss is not always recognized by the patient and vertigo can be attributed to the general symptoms caused by the tumor, a significant number of cases with audiovestibular side effects might stay unrevealed.

In recent years it has become clear that the inner ear is not an immune privileged organ [15,16]. It contains bone-marrow-derived resident macrophages [17,18,19,20], and insults of the inner ear induces the invasion of the cochlea by monocytes and T cells [20,21]. The released proinflammatory cytokines, chemokines, or reactive oxygen species, by the activated immune cells evoke further cellular infiltration [19,22,23]. This cochlear immune system can be involved in either the injury repair processes or the pathogenesis of sensorineural hearing losses [15,16]. Therefore, raising the question of irAEs after the administration of ICIs is well substantiated.

Our aim was to investigate the alterations, if any, caused by PD-1 inhibitor monoclonal antibody treatment in the hearing function and cochlear morphology. To this end, we used a murine model and connected the functional auditory brainstem response (ABR) measurements with histopathological examinations: Cochlear whole-mount dissections to evaluate hair cell numbers, hematoxylin/eosin (HE) staining on mid-modiolar sections to detect changes in spiral ganglion neuron (SGN) numbers and structure, and Iba1 immunohistochemistry to compare the number of activated cochlear macrophages between the treated and control group. We found a preserved function and morphology in the most relevant hearing range (4–32 kHz) and in the corresponding apical-middle cochlear turns, and an enhanced outer hair cell (OHC) survival in the high-frequency basal turn with a moderate increase in the number of Iba1-positive macrophages.

## 2. Results

### 2.1. Differences in Hearing Thresholds between Treatment Groups Could Not Be Observed after PD-1 Inhibition

**Figure 1:**
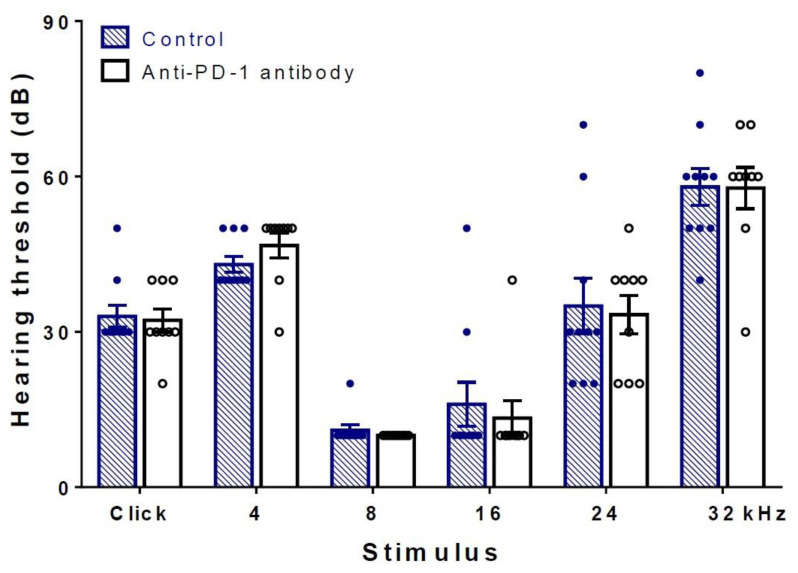
Hearing thresholds of C57BL/6J mice were not influenced by anti-PD-1 antibody treatment. Auditory brainstem response (ABR) measurements were carried out at the end of the 4-week long treatment period (see flowchart in Figure 1 for details). No difference in hearing thresholds was detected either in click or in tone burst stimuli in the 4–32 kHz frequency range between the anti-PD-1 antibody (*n* = 9) and the control group (*n* = 10). Data represent mean ± SEM. Two-way ANOVA followed by Bonferroni post-hoc test; *p* < 0.05 was considered statistically significant (see Section 4).

To investigate whether anti-PD-1 antibody therapy has an effect on the function of hearing, hearing thresholds of isotype control, anti-PD-1 antibody-treated mice were determined and compared at the end of the treatment period. There were no statistically significant differences either by broadband click or frequency-specific pure tones at frequencies 4–32 kHz (Figure 1). Both groups showed hearing performance characteristic for this age of C57BL/6J mice [24].

### 2.2. Anti-PD-1 Antibody Treatment Did Not Affect the Number of Hair Cells in the Apical and Middle Cochlear Turns, but Preserved OHCs in the Basal Turn

**Figure 2:**
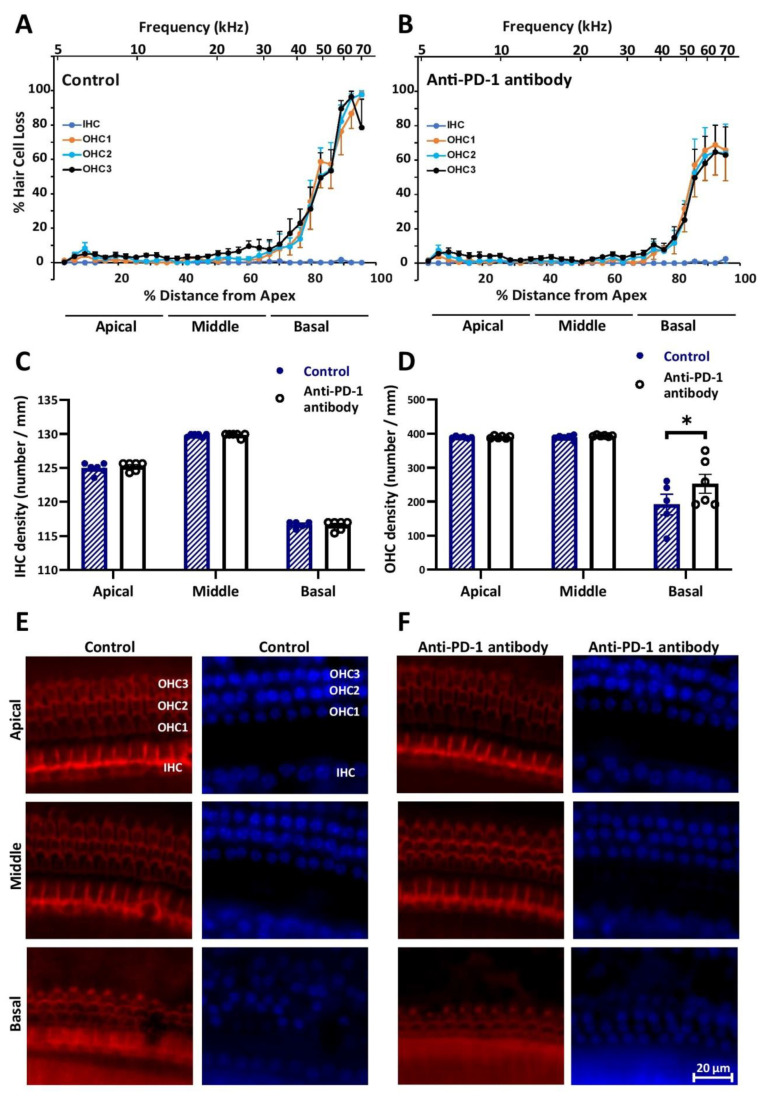
Anti-PD-1 antibody treatment did not change the number of hair cells in the apical and middle turns, but mitigated the loss of outer hair cells (OHCs) in the basal cochlear turns in C57BL/6J mice. Cytocochleograms of the control (**A**) and the anti-PD-1 antibody treated (**B**) group (*n* = 6–6) show the lack of inner hair cell (IHC) and OHC loss in the whole length of the cochlear duct and in the <32 kHz range, respectively. The loss of OHCs in the high-frequency basal turn was less prominent in the anti-PD-1 antibody-treated group. Bar graphs of inner (**C**) and outer (**D**) hair cell densities in the three cochlear segments demonstrate the significant difference in OHC in the basal turn. (**E**,**F**) Representative images of whole-mount dissections of the organ of Corti stained with Alexa Fluor 594 Phalloidin (red) and 4′,6-diamidino-2-phenylindole (DAPI) (blue). Distance from apex was correlated with hearing frequency by using the frequency–place equation by Müller et al., (2004). IHC, inner hair cell, OHC1/2/3: First, second, and third rows of outer hair cells. The scale bar indicates the magnification. Data represents mean ± SEM. Two-way ANOVA followed by Bonferroni post-hoc test; * *p* < 0.05 was considered statistically significant (see Section 4).

Cochleograms, plotted with the evaluation of the staining of actin filaments in inner and outer HCs by Alexa Fluor 594 Phalloidin and counterstaining of nuclei with 4′,6-diamidino-2-phenylindole (DAPI), showed that there is no hair cell loss in the ≤32 kHz frequency range in either treatment groups. However, anti-PD-1 antibody treatment mitigated the loss of OHCs at frequencies higher than 32 kHz (Figure 2A,B,E,F). Inner hair cell (IHCs) loss was undetectable in the basal turn, as well, in both treated groups. Statistical analysis, based on hair cell densities in the apical/middle/basal subdivisions of the tonotopic axis of the cochleae, proved that the difference in OHC density between the control and the anti-PD-1 antibody-treated groups is significant (Figure 2C,D).

### 2.3. Number of SGNs Was Not Changed by Anti-PD-1 Antibody Treatment in Either Cochlear Turn

**Figure 3:**
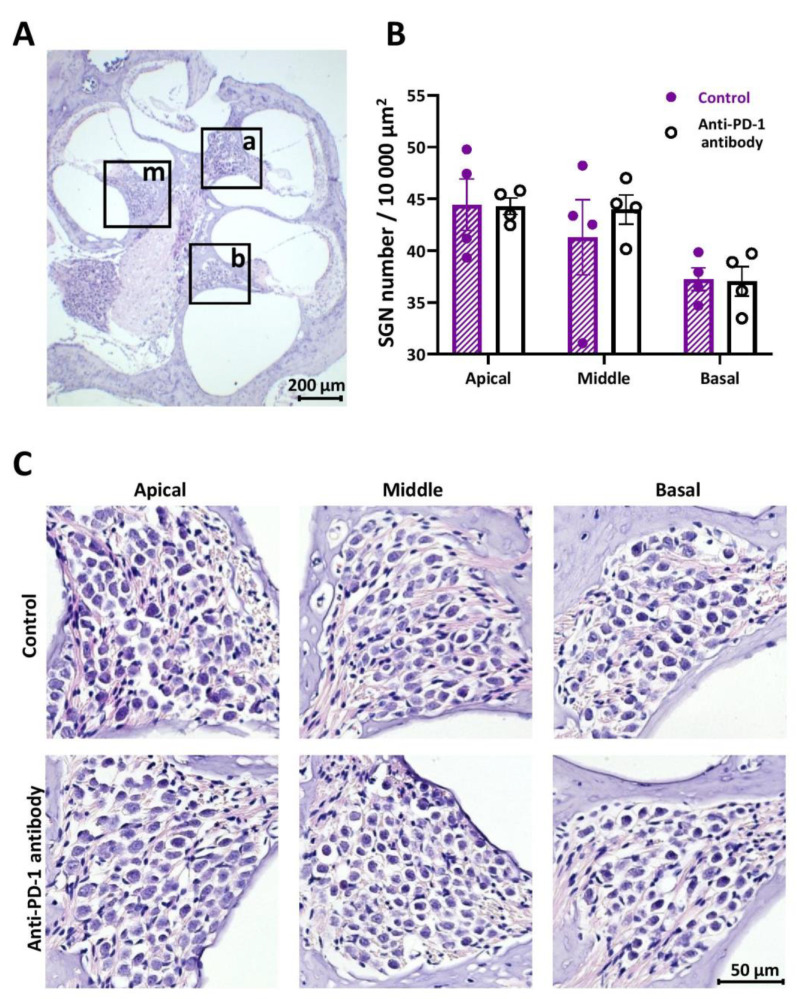
Hematoxylin/eosin (HE) staining of the spiral ganglia. (**A**) Representative image of a HE-stained mid-modiolar section of a cochlea with a 6.3× objective. The squares indicate the spiral ganglia in the apical (a), middle (m), and basal (b) turns. (**B**) Statistical analysis has shown no significant difference between the spiral ganglion neuron (SGN) numbers in the control and anti-PD-1 antibody treated animals (*n* = 4–4 mice, 5 sections each). (**C**) Representative images of the spiral ganglia in the apical, middle, and basal turns demonstrate the lack of difference in SGN numbers and morphology between the control and anti-PD-1 antibody treatment groups (40× objective). The scale bar indicates the magnification. Data represents mean ± SEM. Two-way ANOVA followed by Bonferroni post-hoc test; *p* < 0.05 was considered statistically significant (see Section 4).

There was no alteration in SGN number and morphology after anti-PD-1 antibody treatment in either the apical, middle, or the basal cochlear turns on HE staining of cochlear sections (Figure 3).

### 2.4. PD-1 Inhibition Did Not Affect the Number of Iba-1 Positive Macrophages in the Apical and Middle Cochlear Turns, but Increased It in the Basal Turn

**Figure 4:**
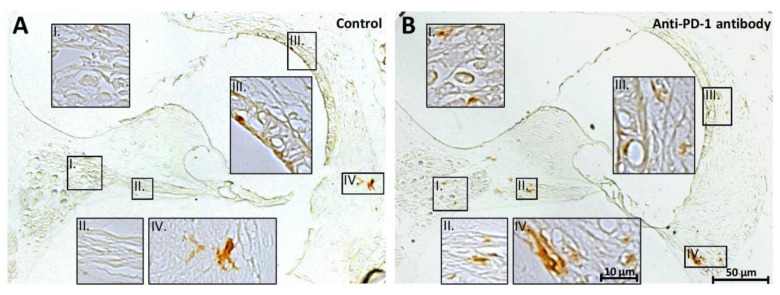
Immunohistochemical staining of the basal cochlear turn with anti-mouse-Iba1 antibody. The macrophage-specific Iba1 staining shows a moderately higher intensity 3,3′-diaminobenzidine DAB staining (i.e., higher macrophage signal) in the anti-PD-1 antibody-treated than in the control animals (representative sections from control (**A**) and anti-PD-1 antibody-treated (**B**) animals). The number of Iba1-positive cells was 1.56 times higher in the basal cochlear turn of the ICI-treated mice (control, *n* = 2; anti-PD-1 antibody-treated, *n* = 3). Roman numerals indicate enlarged areas of the Rosenthal’s canal (I), the peripheral processes of the SGNs (II), the stria vascularis (III), and the spiral ligament (IV). The details of the staining procedure are described in the Section 4.

Iba1-positive macrophages were detected mainly in the Rosenthal’s canal, along the peripheral processes of the SGNs, in the stria vascularis and the spiral ligament as presented in Figure 4. The number of Iba1-stained cells was similar in the control and anti-PD-1 antibody-treated group in the apical and middle cochlear turns (ratio of the cell number in treated/control group were 1.05 and 0.96, respectively). However, in the basal turns, the number of Iba1-positive cells was 1.56 times higher in the anti-PD-1 antibody-treated group (Figure 4).

## 3. Discussion

The role of the immune system in hearing and hearing losses is in the focus of auditory research [19,25,26]. Presence of immune cells (e.g., resident macrophages, infiltrating monocytes) [25,27,28] and their activation contributes fundamentally to the maintenance of cochlear homeostasis and function [21,26,28,29,30,31,32]. However, overactivation of the immune responses can cause cochlear tissue damage and hearing impairment. Certain medications used in clinical practice (e.g., platinum antitumor compounds or aminoglycoside antibiotics)—similarly to cochlear aging and noise exposure—triggers inflammation [16,23,33,34,35,36,37,38,39,40] that results in the development of drug-induced hearing loss [34,41,42,43]. As a consequence, the immunostimulatory PD-1 inhibitors may potentially have harmful effects on hearing as it has been reported in some case studies [12,13,14].

In our mouse model the 4-week long mouse-specific anti-PD-1 therapy did not affect the function of hearing in the 4–32 kHz frequency range and the number of IHCs and OHCs in the apical-middle cochlear turns (<32 kHz). This is in accordance with a murine study by Spielbauer et al. who found that the PD-1 inhibitor monotherapy had no impact on hearing thresholds between 8–40 kHz and on hair cell numbers in the range of 8–32 kHz. However, the Spielbauer study did not investigate the effect of PD-1 inhibition on SGNs and cochlear immune response (e.g., macrophage activation), on the numbers of hair cells in the high frequency regions (>32 kHz) and did not use isotype antibody for control [44]. Kuzucu et al. has found slight and temporary elevation in hearing threshold after anti-PD-1 treatment [45]. Their observation of moderate loss of OHCs was not based on quantitative data or standard methods like cochleogram plotting [46,47,48], and the tonotopy was disregarded as well.

On the other hand, these ABR results are in contrast with the observations of three case reports, where sudden bilateral hearing loss was detected at high frequencies following PD-1 inhibitor treatment [12,13,14]. Furthermore, there are 14 reported cases of sudden hearing loss suspected to result from the use of PD-1 inhibitors in the FDA Adverse Event Reporting System Public Dashboard. Fifty serious and 6 non-serious ear and labyrinth disorders are reported in connection with Keytruda (pembrolizumab) in the EudraVigilance database of the European Medicines Agency. However, these reports do not establish factual causation between the treatment and the symptoms. Clinical studies evaluating the impact of PD-1 inhibitors on the audiovestibular system have not been performed yet.

Immune activation in the cochlea may also be associated with SGN injury [49,50]. The lacking effect of anti-PD-1 therapy on the SGN number and morphology along the tonotopic axis of the cochlear duct in our experiments corresponds well with our measurement on the hearing function. According to our best knowledge, there are no data on the effect of immune checkpoint inhibitor therapy on SGNs we could compare to.

The C57BL/6J strain shows age-related hearing impairment with an increase in hearing thresholds and OHC loss expanding from basal part of the cochlea toward the apical region [51,52]. Loss of IHCs occurs later and progresses much more slowly [52]. In our experiments, whilst IHC loss was negligible along the whole cochlear duct, the age-related loss of OHCs in the basal turn was alleviated by anti-PD-1 administration, suggesting a moderate protection of age-related OHC loss at the high frequency part of the tonotopic axis. We have not found data in the literature on the effect of immune checkpoint inhibitors on hair cells in the >32 kHz frequency range, i.e., in the basal cochlear turn either. This preservation did not appear in our ABR results, because those measurements were not performed above 32 kHz due to the elevated baseline thresholds of C57BL/6 mice at high frequencies [53,54].

The observed OHC preservation in the high frequency range of the cochleogram (>32 kHz) was accompanied with the increased number of Iba-1-positive macrophages exclusively in the basal cochlear turn after anti-PD-1 treatment. According to the literature, macrophages from different cochlear turns show different phenotypes and response patterns to hair cell degeneration in age-related hearing loss (AHL) [27] and only the basal macrophages displayed marked activation of antigen-presenting function for acoustic trauma [20].

The enhanced macrophage activity may be responsible for the mitigated OHC loss in the basal turn with mechanisms similar to those that reduced pathology and improved memory in the Alzheimer’s disease (AD) murine model after a PD-1 immune checkpoint blockade. AHL is partly contributed to chronic, low-grade inflammation in the cochlea, consisting of failure in downregulation of proinflammatory proteins and accumulation of cell debris resulting from insufficient elimination. In AD, amyloid-β (Aβ) accumulation leads to a chronic stimulation of the immune system which initiates a vicious circle: Cytokines such as interleukin 1-β, IL-6, and tumor necrosis factor-α stimulate the synthesis of β-amyloid precursor protein and Aβ peptides [55]. The blockade of the PD-1 pathway results in an increased systemic IFN-γ-dependent immune response that facilitates the entry of monocyte-derived macrophages to the brain, thus enhancing the elimination of Aβ plaques [56,57,58]. We assume that our findings of mitigated outer hair cell loss in the basal cochlear turn following ICI treatment and a simultaneous increased macrophage Iba1-immunoreactivity in the corresponding region can be a consequence of a similar mechanism: Stimulated immune response might help cleaning accumulated cell debris, thus limiting chronic inflammation which results in more preserved cellular integrity of the organ of Corti.

Despite the fact that overactivation of the immune system is involved in the pathology of sensorineural hearing losses, the revolutionary anti-PD-1 therapy with an immunostimulatory effect does not seem to have a significant impact on the functional and morphological integrity of the inner ear in the most relevant hearing range. The immune blockade of PD-1 does not lead to hearing loss or worsening of the pre-existing hearing impairment in the apical-middle turns (<32 kHz). Moreover, a noticeable preservation of OHCs appears in the high frequency (>32 kHz) basal part of the cochlea. Our findings also demonstrate the Janus face of immuno-pharmacotherapy.

## 4. Materials and Methods

### 4.1. Experimental Animals

Experimental procedures were carried out on male C57BL/6J mice (Oncological Research Center, Department of Experimental Pharmacology, H-1122 Budapest, Hungary), ranging from 8 to 10 weeks of age at the beginning of the study. Mice were maintained under a 12:12 h light–dark cycle under controlled environmental conditions (20–24 °C and 35–75% relative humidity) in individually ventilated cages with shelters, holding 2–3 animals per cage. Standard rodent chow and tap water were provided ad libitum throughout the entire duration of the experiment. All procedures were approved by the National Scientific Ethical Committee on Animal Experimentation and the Semmelweis University’s Institutional Animal Care and Use Committee (H-1089 Budapest, Hungary) in accordance with NIH guidelines (National Research Council (2011), Guide for the Care and Use of Laboratory Animals: Eighth Edition) and permitted by the government of Food Chain Safety and Animal Health Directorate of the Government Office for Pest County (project identification code: PE/EA/1912-7/2017; date of approval: November 2017).

### 4.2. Experimental Groups and Drug Administration

**Figure 5:**
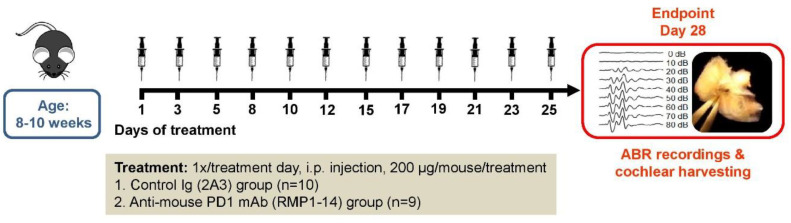
Flowchart of the experiments. C57BL/6J mice received either an anti-mouse PD-1 (anti-PD-1 antibody) or an IgG2a isotype antibody (control) injection intraperitoneally three days per week for 4 weeks with a 2–3 days lag time between treatments. Syringes indicate the application of antibodies at the dose of 200 µg/mouse/treatment. Measuring the function of hearing by auditory evoked potentials (ABR) and harvesting of cochlear tissues for morphological analysis were performed at the end of the treatment period.

Twenty C57BL/6J mice were randomly assigned to either a control (*n* = 10) or an anti-PD-1 antibody-treated group (*n* = 9, one animal died during the experiment). Purified *InVivo*MAb rat IgG2a isotype control antibody (clone 2A3), and in addition, *InVivo*MAb anti-mouse PD-1 antibody (clone RMP1-14), used for immune checkpoint blockade were purchased from BioXCell. Drugs were administered once on the day of treatments by intraperitoneal (i.p.) injections at a dose of 200 μg/mouse/treatment. Antibodies were delivered to mice three times per week over the treatment period lasting for 4 weeks (Figure 5). Treatment schedule was based on previous publications assessing the anti-tumor efficacy and side-effect profile of this particular antibody clone [59,60,61,62].

### 4.3. Hearing Assessment

ABR audiometry was performed at the end of the treatment period to test auditory function by measuring hearing thresholds at different frequencies [63]. Briefly, evoked potentials were recorded in the right ear of each mouse under ketamine/xylazine anesthesia (100 and 10 mg/kg, i.p., respectively). Body temperature of mice was maintained at 36–38 °C with a temperature-controlled heating pad (Supertech Instruments, H-7624 Pécs, Hungary). ABR waves were recorded with subdermal needle electrodes placed at the vertex (active electrode), behind the right pinna (reference electrode), and at the rear leg (ground). Measurements were carried out in a sound-proof chamber using a TDT workstation (TDT system 3 with RX6 signal processor and RA16 Medusa Base Station; Tucker–Davis Technologies (TDT), Alachua, FL, USA). Click (0.4 ms duration) and tone burst (3 ms duration, 0.2 ms rise/decay, frequency range 4–32 kHz) stimuli were generated in the SigGenRP software package and delivered in a closed acoustic system using EC-1 electrostatic speaker, controlled by the BioSigRP software. Digital signals were amplified by RA4PA Medusa PreAmplifier connected to RA4LI Low Impedance Headstage (TDT, Alachua, FL, USA). Sound pressure levels of the click stimulus were increased in 10-dB steps from 0 to 80 dB, whilst intensity was attenuated in 10 dB steps from 90 to 10 dB in tone burst stimulation mode controlled by a PA5 Programmable Attenuator (TDT, Alachua, FL, USA). For calibrating the sound delivery system, a half-inch free field preamplifier integrated microphone was used (ACO Pacific Inc., Belmont, CA 94002, USA; Model 7017) with the application of the SigCalRP calibration software. Evoked potentials were amplified, filtered, and averaged 800 times in real-time. The hearing threshold was defined as the minimal intensity level at which an ABR waveform with an identifiable peak could be detected visually.

### 4.4. Tissue Preparation

The 10 control and 9 anti-PD-1 antibody-treated mice were euthanized, and both right and left inner ears were dissected from the temporal bone. After removing the stapes and carefully performing two openings on the cochleae—one in the apex and one in the round window—they were perfused with 0.2 mL 4% paraformaldehyde (PFA) in phosphate-buffered saline (PBS; pH = 7.4), then immersed in the 4% PFA for 2 h at room temperature in Eppendorf tubes on a rocking shaker. Cochleae were then decalcified in 5% EDTA in PBS with 0.4% PFA in 15-mL centrifuge tubes for 6 days at room temperature on a rocking shaker. The EDTA solution over the cochleae was changed daily.

### 4.5. Whole-Mount Cochlear Dissection and Phalloidin/DAPI Staining

The organ of Corti was removed from the decalcified right cochleae of 6 control and 6 anti-PD-1 antibody-treated animals under stereomicroscope using microsurgical instruments. The bony wall, the vestibular system, spiral ligament, modiolus, and tectorial membrane were carefully removed, and the basilar membrane was cut into 3–6 longitudinal segments. The samples were rinsed three times in PBS for 5 min each time, then immersed in 0.3% Triton X-100 in PBS for 10 min and incubated with Alexa Fluor 594 Phalloidin (1:200, Thermo Fisher Scientific, Waltham, MA, USA) for 40 min at room temperature. After washing 3 times with PBS for 5 min, nuclei were stained with DAPI (1 µmol/L, 5 min, Cell Signaling Technology, Leiden, The Netherlands). Subsequently, samples were washed 3 times in PBS for 5 min. Finally, cochlear turns were placed on a glass slide using Prolong Gold antifade reagent (Thermo Fisher Scientific, Waltham, MA, USA).

### 4.6. Cytocochleogram Plotting

Hair cells were imaged in the epifluorescent mode of a Leica LMD6 microscope coupled to a CCD camera (DFC7000T; Leica Biosystems, Wetzlar, Germany) using a 40× HC PL Fluotar objective (Leica Biosystems, Wetzlar, Germany). Cells were counted manually. In every 190–250 µm segment alongside the pillar cells the number of missing hair cells was counted. Hair cell counts were analyzed using a customized version of the Kresge Hearing Research Institute CytoGram Program, Version 3.0.6 (Ann Arbor, MI, USA). An average of 95% of the total length of the basilar membrane from the apex was evaluated. In both the control (*n* = 6) and anti-PD-1-treated groups (*n* = 6), cochleograms were plotted. The frequency-place equation by Müller et al. (2004) was used to correlate the location to frequency. For statistical analysis, the evaluated section of the basilar membrane was divided into three equal segments in length: apical, middle, and basal.

### 4.7. Modiolar Sectioning for Hematoxylin-Eosin Staining and Immunohistochemistry

A graded series of ethanol solutions (35, 50, 70%, 2 × 15 min each) was used to dehydrate the decalcified tissues. Further dehydration with a graded series of ethanol (70, 80, 95% and absolute ethanol, each of them 2 × 1 h) and xylol treatment (2 × 1 h) were performed in a Leica TP 1020 tissue processor (Leica Biosystems, Wetzlar, Germany). Ultimately, the cochleae were embedded in 60 °C paraffin, and 4-µm sections were cut with a Leica HistoCore MULTICUT microtome (Leica Biosystems, Wetzlar, Germany) for hematoxylin/eosin (HE) and Iba1 staining.

### 4.8. Hematoxylin/Eosin (HE) Staining and Counting Spiral Ganglion Neurons (SGNs)

To avoid repeated counting of the same SGNs, every fourth section was stained with HE (Harris Hematoxylin: Sigma–Aldrich, St. Louis, MO, USA; Eosin: Leica Biosystems, Wetzlar, Germany). Images of the Rosenthal’s canal were acquired in the brightfield mode of a Leica LMD6 microscope (Leica Microsystems, Wetzlar, Germany) using a 40× HC PL Fluotar objective (Leica Microsystems, Wetzlar, Germany). SGNs were counted in a representative area (16,785 µm^2^ in average, but min. 6000 µm^2^) in four anti-PD-1-treated (2 left and 2 right ears) and four control (2 left and 2 right ears) animals. Five sections per mouse were evaluated and the average density of SGNs was calculated in the apical, middle, and basal turns.

### 4.9. Iba1 Immunohistochemistry

The 4-µm modiolar sections were deparaffinized and hydrated with xylene and a graded series of ethanol solutions, respectively. Heat-induced antigen retrieval was performed in microwave-heated citrate buffer (pH ≈ 6.5) for a total time of 10 min (4 times 1.5 min and 2 times 2 min, with pauses of the same length in between). The slides were cooled, washed in PBS for 3 × 5 min, then the endogenous peroxidase activity was blocked with 3% H_2_O_2_ in PBS for 10 min. Samples were washed in PBS for 3 × 5 min, blocked in 2.5% normal goat serum (Vector Laboratories, Inc., Burlingame, CA 94010, USA) containing 2.5% non-fat dried milk powder in PBS for 1 h at room temperature, then incubated with the primary antibody (anti-Iba1, 1:2000, Rabbit, FUJIFILM Wako Pure Chemical Corporation, Neuss, Germany) overnight at 4 °C. After washing 3 times for 10 min in PBS, secondary antibody was used SignalStain^®^ Boost IHC Detection Reagent (HRP, Rabbit; Cell Signaling Technology, Leiden, The Netherlands) in accordance with the manufacturer’s instructions, and washed again. The specific signal was developed with ImmPACT DAB EqV Substrate Kit (Vector Laboratories, Inc., Burlingame, CA 94010, USA), dehydrated, and covered by coverslips using CV Mount (Leica Biosystems, Wetzlar, Germany). Iba1 positive cells were evaluated in an observer-blinded fashion by two independent persons in 6 control (from 2 animals) and 6 anti-PD-1 antibody-treated (from 3 animals) 4-µm sections.

### 4.10. Statistics

Data were analyzed with two-way ANOVA followed by a Bonferroni post-hoc comparison test using the 8.4.3 version of GraphPad Prism. Results are indicated as mean ± standard error of the mean (SEM).

## Abbreviations

ICI	immune checkpoint inhibitor
PD-L1	programmed death-ligand 1
PD-1	programmed cell death protein 1
NSCLC	non-small-cell lung cancer
RCC	renal cell carcinoma
irAE	immune-related adverse event
ABR	auditory brainstem response
DAB	3,3′-diaminobenzidine
HE	hematoxylin/eosin
SGN	spiral ganglion neuron
OHC	outer hair cell
IHC	inner hair cell
NIH	National Institutes of Health
PFA	paraformaldehyde
PBS	phosphate-buffered saline
EDTA	ethylenediamine-tetraacetate
DAPI	4′,6-diamidino-2-phenylindole
Iba1	ionized calcium-binding adaptor molecule 1
HRP	horseradish peroxidase
ANOVA	analysis of variance
SEM	standard error of the mean
Aβ	amyloid-β

## References

[1] Morello S., Pinto A., Blandizzi C., Antonioli L. (2016). Myeloid cells in the tumor microenvironment: Role of adenosine. Oncoimmunology.

[2] Freeman G.J., Long A.J., Iwai Y., Bourque K., Chernova T., Nishimura H., Fitz L.J., Malenkovich N., Okazaki T., Byrne M.C. (2000). Engagement of the PD-1 immunoinhibitory receptor by a novel B7 family member leads to negative regulation of lymphocyte activation. J. Exp. Med..

[3] Schreiber R.D., Old L.J., Smyth M.J. (2011). Cancer immunoediting: Integrating immunity’s roles in cancer suppression and promotion. Science.

[4] Topalian S.L., Hodi F.S., Brahmer J.R., Gettinger S.N., Smith D.C., McDermott D.F., Powderly J.D., Carvajal R.D., Sosman J.A., Atkins M.B. (2012). Safety, Activity, and Immune Correlates of Anti–PD-1 Antibody in Cancer. N. Engl. J. Med..

[5] Page D.B., Postow M.A., Callahan M.K., Allison J.P., Wolchok J.D. (2014). Immune Modulation in Cancer with Antibodies. Annu. Rev. Med..

[6] Antonioli L., Yegutkin G.G., Pacher P., Blandizzi C., Haskó G. (2016). Anti-CD73 in cancer immunotherapy: Awakening new opportunities. Trends Cancer.

[7] Brahmer J.R., Drake C.G., Wollner I., Powderly J.D., Picus J., Sharfman W.H., Stankevich E., Pons A., Salay T.M., McMiller T.L. (2010). Phase I study of single-agent anti-programmed death-1 (MDX-1106) in refractory solid tumors: Safety, clinical activity, pharmacodynamics, and immunologic correlates. J. Clin. Oncol..

[8] Plimack E.R., Bellmunt J., Gupta S., Berger R., Chow L.Q.M., Juco J., Lunceford J., Saraf S., Perini R.F., O’Donnell P.H. (2017). Safety and activity of pembrolizumab in patients with locally advanced or metastatic urothelial cancer (KEYNOTE-012): A non-randomised, open-label, phase 1b study. Lancet Oncol..

[9] Postow M.A. (2015). Managing Immune Checkpoint-Blocking Antibody Side Effects. Am. Soc. Clin. Oncol. Educ. B..

[10] Hamid O., Robert C., Daud A., Hodi F.S., Hwu W.-J., Kefford R., Wolchok J.D., Hersey P., Joseph R.W., Weber J.S. (2013). Safety and Tumor Responses with Lambrolizumab (Anti–PD-1) in Melanoma. N. Engl. J. Med..

[11] Antoun J., Titah C., Cochereau I. (2016). Ocular and orbital side-effects of checkpoint inhibitors. Curr. Opin. Oncol..

[12] Zibelman M., Pollak N., Olszanski A.J. (2016). Autoimmune inner ear disease in a melanoma patient treated with pembrolizumab. J. Immunother. Cancer.

[13] Hobelmann K., Fitzgerald D. (2019). A Case of Pembrolizumab Induced Autoimmune Sensorineural Hearing Loss. J. Otol. Rhinol..

[14] Tampio A.J.F., Dhanireddy S., Sivapiragasam A., Nicholas B.D. (2020). Bilateral Sensorineural Hearing Loss Associated With Nivolumab Therapy for Stage IV Malignant Melanoma. Ear Nose Throat J..

[15] Raphael Y. (2002). Cochlear pathology, sensory cell death and regeneration. Br. Med. Bull..

[16] Tan W.J., Thorne P.R., Vlajkovic S.M. (2013). Noise-induced cochlear inflammation. World J. Otorhinolaryngol..

[17] Harris J.P. (1983). Immunology of the inner ear: Response of the inner ear to antigen challenge. Otolaryngol. Neck Surg..

[18] Okano T., Nakagawa T., Kita T., Kada S., Yoshimoto M., Nakahata T., Ito J. (2008). Bone marrow-derived cells expressing Iba1 are constitutively present as resident tissue macrophages in the mouse cochlea. J. Neurosci. Res..

[19] Köles L., Szepesy J., Berekméri E., Zelles T. (2019). Purinergic Signaling and Cochlear Injury-Targeting the Immune System?. Int. J. Mol. Sci..

[20] Yang W., Vethanayagam R.R., Dong Y., Cai Q., Hu B.H. (2015). Activation of the antigen presentation function of mononuclear phagocyte populations associated with the basilar membrane of the cochlea after acoustic overstimulation. Neuroscience.

[21] Hu B.H., Zhang C., Frye M.D. (2018). Immune cells and non-immune cells with immune function in mammalian cochleae. Hear. Res..

[22] Wood M.B., Zuo J. (2017). The Contribution of Immune Infiltrates to Ototoxicity and Cochlear Hair Cell Loss. Front. Cell. Neurosci..

[23] Tan W.J.T., Thorne P.R., Vlajkovic S.M. (2016). Characterisation of cochlear inflammation in mice following acute and chronic noise exposure. Histochem. Cell Biol..

[24] Fujita T., Yamashita D., Uehara N., Inokuchi G., Hasegawa S., Otsuki N., Nibu K.I. (2015). A high-fat diet delays age-related hearing loss progression in C57BL/6J mice. PLoS ONE.

[25] Kalinec G.M., Lomberk G., Urrutia R.A., Kalinec F. (2017). Resolution of Cochlear Inflammation: Novel Target for Preventing or Ameliorating Drug-, Noise- and Age-related Hearing Loss. Front. Cell. Neurosci..

[26] Fujioka M., Okano H., Ogawa K. (2014). Inflammatory and immune responses in the cochlea: Potential therapeutic targets for sensorineural hearing loss. Front. Pharmacol..

[27] Frye M.D., Yang W., Zhang C., Xiong B., Hu B.H. (2017). Dynamic activation of basilar membrane macrophages in response to chronic sensory cell degeneration in aging mouse cochleae. Hear. Res..

[28] Zhang W., Dai M., Fridberger A., Hassan A., DeGagne J., Neng L., Zhang F., He W., Ren T., Trune D. (2012). Perivascular-resident macrophage-like melanocytes in the inner ear are essential for the integrity of the intrastrial fluid-blood barrier. Proc. Natl. Acad. Sci. USA.

[29] Hirose K., Discolo C.M., Keasler J.R., Ransohoff R. (2005). Mononuclear phagocytes migrate into the murine cochlea after acoustic trauma. J. Comp. Neurol..

[30] Koo J.-W., Quintanilla-Dieck L., Jiang M., Liu J., Urdang Z.D., Allensworth J.J., Cross C.P., Li H., Steyger P.S. (2015). Endotoxemia-mediated inflammation potentiates aminoglycoside-induced ototoxicity. Sci. Transl. Med..

[31] Shi X. (2016). Pathophysiology of the cochlear intrastrial fluid-blood barrier (review). Hear. Res..

[32] Antonioli L., Blandizzi C., Pacher P., Guilliams M., Haskó G. (2019). Rethinking Communication in the Immune System: The Quorum Sensing Concept. Trends Immunol..

[33] Verschuur C., Agyemang-Prempeh A., Newman T.A. (2014). Inflammation is associated with a worsening of presbycusis: Evidence from the MRC national study of hearing. Int. J. Audiol..

[34] Kaur T., Mukherjea D., Sheehan K., Jajoo S., Rybak L.P., Ramkumar V. (2011). Short interfering RNA against STAT1 attenuates cisplatin-induced ototoxicity in the rat by suppressing inflammation. Cell Death Dis..

[35] So H., Kim H., Lee J.-H., Park C., Kim Y., Kim E., Kim J.-K., Yun K.-J., Lee K.-M., Lee H.-Y. (2007). Cisplatin cytotoxicity of auditory cells requires secretions of proinflammatory cytokines via activation of ERK and NF-kappaB. J. Assoc. Res. Otolaryngol..

[36] Hirose K., Sato E. (2011). Comparative analysis of combination kanamycin-furosemide versus kanamycin alone in the mouse cochlea. Hear. Res..

[37] Kaur T., Ohlemiller K.K., Warchol M.E. (2018). Genetic disruption of fractalkine signaling leads to enhanced loss of cochlear afferents following ototoxic or acoustic injury. J. Comp. Neurol..

[38] Ladrech S., Wang J., Simonneau L., Puel J.-L., Lenoir M. (2007). Macrophage contribution to the response of the rat organ of Corti to amikacin. J. Neurosci. Res..

[39] Sato E., Shick H.E., Ransohoff R.M., Hirose K. (2010). Expression of Fractalkine Receptor CX3CR1 on Cochlear Macrophages Influences Survival of Hair Cells Following Ototoxic Injury. J. Assoc. Res. Otolaryngol..

[40] Wu W.J., Sha S.H., McLaren J.D., Kawamoto K., Raphael Y., Schacht J. (2001). Aminoglycoside ototoxicity in adult CBA, C57BL and BALB mice and the Sprague-Dawley rat. Hear. Res..

[41] Cianfrone G., Pentangelo D., Cianfrone F., Mazzei F., Turchetta R., Orlando M.P., Altissimi G. (2011). Pharmacological drugs inducing ototoxicity, vestibular symptoms and tinnitus: A reasoned and updated guide. Eur. Rev. Med. Pharmacol. Sci..

[42] Oh G.-S., Kim H.-J., Choi J.-H., Shen A., Kim C.-H., Kim S.-J., Shin S.-R., Hong S.-H., Kim Y., Park C. (2011). Activation of Lipopolysaccharide-TLR4 Signaling Accelerates the Ototoxic Potential of Cisplatin in Mice. J. Immunol..

[43] Guthrie O.W. (2008). Aminoglycoside induced ototoxicity. Toxicology.

[44] Spielbauer K., Cunningham L., Schmitt N. (2018). PD-1 Inhibition Minimally Affects Cisplatin-Induced Toxicities in a Murine Model. Otolaryngol. Head Neck Surg..

[45] Kuzucu İ., Baklacı D., Guler İ., Uçaryılmaz E.Ö., Kum R.O., Özcan M. (2019). Investigation of the Ototoxic Effect of Pembrolizumab Using a Rat Model. Cureus.

[46] Viberg A., Canlon B. (2004). The guide to plotting a cochleogram. Hear. Res..

[47] Bohne B.A., Harding G.W. (1993). Combined organ of Corti/modiolus technique for preparing mammalian cochleas for quantitative microscopy. Hear. Res..

[48] Raphael Y., Altschuler R.A. (1991). Scar formation after drug-induced cochlear insult. Hear. Res..

[49] Kaur T., Zamani D., Tong L., Rubel E.W., Ohlemiller K.K., Hirose K., Warchol M.E. (2015). Fractalkine Signaling Regulates Macrophage Recruitment into the Cochlea and Promotes the Survival of Spiral Ganglion Neurons after Selective Hair Cell Lesion. J. Neurosci..

[50] Sekiya T., Tanaka M., Shimamura N., Suzuki S. (2001). Macrophage invasion into injured cochlear nerve and its modification by methylprednisolone. Brain Res..

[51] Zheng Q.Y., Johnson K.R., Erway L.C. (1999). Assessment of hearing in 80 inbred strains of mice by ABR threshold analyses. Hear. Res..

[52] Spongr V.P., Flood D.G., Frisina R.D., Salvi R.J. (1997). Quantitative measures of hair cell loss in CBA and C57BL/6 mice throughout their life spans. J. Acoust. Soc. Am..

[53] Ohlemiller K.K., Wright J.S., Heidbreder A.F. (2000). Vulnerability to noise-induced hearing loss in “middle-aged” and young adult mice: A dose-response approach in CBA, C57BL, and BALB inbred strains. Hear. Res..

[54] Keithley E.M., Canto C., Zheng Q.Y., Fischel-Ghodsian N., Johnson K.R. (2004). Age-related hearing loss and the ahl locus in mice. Hear. Res..

[55] Blasko I., Stampfer-Kountchev M., Robatscher P., Veerhuis R., Eikelenboom P., Grubeck-Loebenstein B. (2004). How chronic inflammation can affect the brain and support the development of Alzheimer’s disease in old age: The role of microglia and astrocytes. Aging Cell.

[56] Schwartz M. (2017). Can immunotherapy treat neurodegeneration?. Science.

[57] Baruch K., Deczkowska A., Rosenzweig N., Tsitsou-Kampeli A., Sharif A.M., Matcovitch-Natan O., Kertser A., David E., Amit I., Schwartz M. (2016). PD-1 immune checkpoint blockade reduces pathology and improves memory in mouse models of Alzheimer’s disease. Nat. Med..

[58] Rosenzweig N., Dvir-Szternfeld R., Tsitsou-Kampeli A., Keren-Shaul H., Ben-Yehuda H., Weill-Raynal P., Cahalon L., Kertser A., Baruch K., Amit I. (2019). PD-1/PD-L1 checkpoint blockade harnesses monocyte-derived macrophages to combat cognitive impairment in a tauopathy mouse model. Nat. Commun..

[59] Martini E., Kunderfranco P., Peano C., Carullo P., Cremonesi M., Schorn T., Carriero R., Termanini A., Colombo F.S., Jachetti E. (2019). Single-Cell Sequencing of Mouse Heart Immune Infiltrate in Pressure Overload-Driven Heart Failure Reveals Extent of Immune Activation. Circulation.

[60] Holmgaard R.B., Zamarin D., Munn D.H., Wolchok J.D., Allison J.P. (2013). Indoleamine 2,3-dioxygenase is a critical resistance mechanism in antitumor T cell immunotherapy targeting CTLA-4. J. Exp. Med..

[61] Twyman-Saint Victor C., Rech A.J., Maity A., Rengan R., Pauken K.E., Stelekati E., Benci J.L., Xu B., Dada H., Odorizzi P.M. (2015). Radiation and dual checkpoint blockade activate non-redundant immune mechanisms in cancer. Nature.

[62] Moynihan K.D., Opel C.F., Szeto G.L., Tzeng A., Zhu E.F., Engreitz J.M., Williams R.T., Rakhra K., Zhang M.H., Rothschilds A.M. (2016). Eradication of large established tumors in mice by combination immunotherapy that engages innate and adaptive immune responses. Nat. Med..

[63] Polony G., Humli V., Andó R., Aller M., Horváth T., Harnos A., Tamás L., Vizi E.S., Zelles T. (2014). Protective effect of rasagiline in aminoglycoside ototoxicity. Neuroscience.

